# Oral Lesions Following Anti-SARS-CoV-2 Vaccination: A Systematic Review

**DOI:** 10.3390/ijerph191610228

**Published:** 2022-08-17

**Authors:** Federica Di Spirito, Alessandra Amato, Maria Pia Di Palo, Maria Contaldo, Francesco D’Ambrosio, Roberto Lo Giudice, Massimo Amato

**Affiliations:** 1 Department of Medicine, Surgery and Dentistry, University of Salerno, 84084 Salerno, Italy; 2 Department of Neuroscience, Reproductive Science and Dentistry, University of Naples Federico II, 80138 Naples, Italy; 3 Multidisciplinary Department of Medical-Surgical and Odontostomatological Specialities, University of Campania “Luigi Vanvitelli”, 81100 Naples, Italy; 4 Department of Human Pathology in Adulthood and Childhood “G. Barresi”, University Hospital “G. Martino” of Messina, Via Consolare Valeria 1, 98123 Messina, Italy

**Keywords:** SARS-CoV-2, coronavirus disease 2019, COVID-19, oral lesions, vaccine, vaccination

## Abstract

Increasing evidence relate anti-SARS-CoV-2 vaccinations to orofacial adverse reactions, therefore, the present systematic review aimed to evaluate primary oral lesions diagnosed in adult subjects, following the WHO Emergency Use Listing approved and EMA authorized vaccines, also in relation to cases’ age, gender, comorbidities, and history of COVID-19, and in relation to vaccine type and doses. The study protocol, registered on PROSPERO (CRD42022339032) and compliant with the PRISMA statement, included an electronic search across Scopus, MEDLINE/PubMed, BioMed Central databases, and PROSPERO, ended on 18 June 2022 and succeeded by a manual search, an independent data extraction, and arisk of bias evaluation through ROBINS-I tool. Qualitatively synthesized data from the 13studies included showed an overall low prevalence (16 cases), though higher in females (68.8%), of oral lesions, mainly erosions and ulcers (34.5%). Nine cases were diagnosed following Pfizer-BioNTech, two Moderna, and one AstraZeneca, Serum Institute of India, Sinopharm, and Johnson&Johnson vaccines, respectively; specifically, eight after the first dose and seven after the second. In one case, vaccine type and dose were not specified. Considering newly developing vaccines, presented findings may be updated and further studies needed to highlight factors affecting oral lesion occurrence and specific macro-microscopic phenotypes in relation to cases’ and vaccines’ characteristics.

## 1. Introduction

Adverse drug reactions (ADRs) are potentially dangerous sequelae related to the use of medicinal products [1,2], including vaccines [3]. Mucocutaneous ADRs, potentially involving the oral cavity, have been previously reported following vaccines against several viruses (hepatitisB, influenza, measles, mumps, and rubella) and bacteria (Clostridium tetani, Corynebacterium diphtheria, and Bordetella pertussis) [3,4,5]. Moreover, orofacial ADRs, such as temporary one-sided facial drooping and tongue, face, or throat swelling, have also been described following anti-SARS-CoV-2 vaccines, along with skin and oral lesions [3,4,5].

Anti-SARS-CoV-2 vaccines are considered essential tools in managing the COVID-19 pandemic [6], significantly reducing the risk of viral transmission and infection, preventing severe forms of COVID-19 [7], and decreasing hospitalization and death rates [6,8]. However, the exceptional circumstances encountered in recent years, due to the global spread of SARS-CoV-2 and related vaccination campaigns, have rekindled interest in the potential risks connected to vaccine administration [9]. Consequently, it seems particularly relevant to address such an issue to promptly manage adverse events and to improve patient outcomes [3], especially considering that vaccinations prevent 2 to 3 million yearly deaths from infectious diseases [3].

Therefore, the present systematic review primarily aimed to assess current data on cases diagnosed with oral lesions among adult subjects (≥18 years old) who had received at least one dose of the World Health Organization (WHO) Emergency Use Listing approved [10] and European Medicines Agency (EMA) authorized [11] anti-SARS-CoV-2 vaccines, classify oral mucosal manifestations based on primary oral lesions, and describe their macroscopic and microscopic features. The secondary aim of the study was to evaluate reported oral lesions in relation to cases’ demographic characteristics, comorbidities, ongoing treatments, history of COVID-19 and to vaccine type and doses administered to highlight putative factors affecting adverse reaction occurrence.

## 2. Materials and Methods

The study protocol, registered on the PROSPERO systematic review register (registration number: CRD42022339032), was developed before starting the literature search and data analysis and performed under the Preferred Reporting Items for Systematic Reviews and Meta-analyses (PRISMA) statement [12,13].

Question formulation, search strategies definition, and study selection criteria were developed according to the PICO model [14]. The study question is “Are anti-SARS-CoV-2 vaccines associated with the occurrence of oral mucosal lesions?” focusing on:

P—Population: adult (≥18 years) subjects who received at least one dose of the anti-SARS-CoV-2 vaccine;

I—Intervention: WHO Emergency Use Listing approved and EMA authorized anti-SARS-CoV-2 vaccines; specifically mRNA BNT162b2 Comirnaty (Pfizer-BioNTech—New York, NY, USA and Magonza, Germay); mRNA-1273 Spikevax (Moderna—Cambridge, MA, USA); Vaxzevria ChAdOx1-S (AstraZeneca—Cambridge, UK); ChAdOx1 nCoV-19 Covishield (Serum Institute of India—Pune, India); Covilo/BBIBP-Corv (Sinopharm Beijing—Beijing, China); and Ad26.COV2.S (Johnson&Johnson—New Brunswick, NJ, USA);

C—Comparison: adult (≥18 years) subjects who did not receive any dose of anti-SARS-CoV-2 vaccine;

O—Outcome(s): oral mucosal lesions following anti-SARS-CoV-2 vaccine administration.

### 2.1. Search Strategy

An electronic search was conducted for records published/in press in the English language on Scopus, MEDLINE/PubMed, and BioMed Central databases, and on the PROSPERO register, through 18 June 2022, not applying restrictions concerning date or publication status, and employing the following keywords: 1. Oral lesion OR Oral manifestation AND 2. Vaccine OR Vaccination AND 3. SARS-CoV-2 OR COVID-19.

### 2.2. Study Selection Process

Study selection was independently conducted by three reviewers (FDS, MPDP, and FDA) based on the inclusion/exclusion criteria, and disagreements were solved by a discussion with a fourth author (AA).

Data from relevant prospective, retrospective, and case-control studies, along with case series, case reports, and letters to the editor, describing oral lesions in adult (≥18 years) subjects who received at least one dose of the WHO Emergency Use Listing approved [10] and EMA authorized [11] anti-SARS-CoV-2 vaccines were considered eligible for the present systematic review.

In vitro and pre-clinical in vivo studies, narrative and systematic reviews, conference papers, oral communications, books and chapters, and studies reporting oral outcomes following other anti-SARS-CoV-2 vaccines (not WHO Emergency Use Listing approved and EMA authorized ones) were excluded. Pre-existing and self-reported lesions of the oral mucosa, other orofacial manifestations following anti-SARS-CoV-2 vaccines, and oral lesions in SARS-CoV-2 positive subjects were not currently considered.

After elimination of duplicates, all title-abstracts obtained from the electronic search were screened. For those considered potentially relevant and compliant with the inclusion/exclusion criteria, full-texts were obtained, and, in case of missing data, an attempt to contact Authors was conducted, preliminary to full-text reading.

### 2.3. Data Extraction and Collection

Data extraction and collection were performed on a dedicated form, developed following the models proposed for intervention reviews on RCTs and non-RCTs [15], by two independent reviewers (MPDP, FDS), and involving a third reviewer (AA) if needed.

For each study included in the present systematic review, the following data were retrieved: author(s), study design and funding, year and journal of publication; investigated population’s sample size, gender, mean age, comorbidities, and related pharmacological therapies, history of COVID-19, anti-SARS-CoV-2 vaccine type and doses administered, the time between administration and oral lesion onset; oral lesions macroscopic and microscopic features, diagnostic procedure, definitive diagnosis, and treatment(s).

### 2.4. Data Synthesis

A narrative synthesis was performed, focusing on the investigated population, intervention, comparison, and outcome.

Data from included studies were qualitatively synthesized through descriptive statistical analyses using Microsoft Excel software 2019 (Microsoft Corporation, Redmond, WA, USA). Primary oral lesions following anti-SARS-CoV-2 vaccination were categorized according to a previously proposed classification [16] and classified as erosions and ulcers (aphthous-like/erythema multiforme/herpetiform patterned), plaques (white/red), vesicles and bullae, and maculae and petechiae.

### 2.5. Quality Assessment

The risk of bias of the nonrandomized studies included in the present systematic review was assessed by three independent reviewers (FDS, MPDP, FDA) through the ROBINS-I (Risk Of Bias In Non-randomized Studies of Interventions) [17] tool, which analyzes seven domains of bias: confounders, selection of participants, classification of interventions, deviation of planned interventions, measurement of results, missing data, and selection of reported results.

Accordingly, low and moderate risk of bias was defined when the study was judged to be at low or moderate risk of bias for all domains; serious or critical risk of bias was defined in case of a serious or critical risk of bias for at least one domain, respectively.

## 3. Results

### 3.1. Study Selection

A total of 681 records were obtained from the electronic search, specifically 199 from MEDLINE/PubMed, 158 from Scopus, 322 from BioMed Central databases, and 2 from the PROSPERO register; subsequently, 396 duplicates were eliminated.

The remaining 285 title-abstracts were screened, and 267 were excluded; of the 18 abstracts relevant to and compliant with the eligibility criteria, full-texts were assessed. Additionally, 10 articles were excluded, specifically because 4 studies described oral manifestations not following anti-SARS-CoV-2 vaccination; 3 did not report lesions of the oral mucosa; 1 involved a <18 years old subject; 1 reported oral lesions not diagnosed by clinical examination but through questionnaires administration; and 1 was a systematic review of the literature.

In total, eight studies were included in the present systematic review.

**Figure 1:**
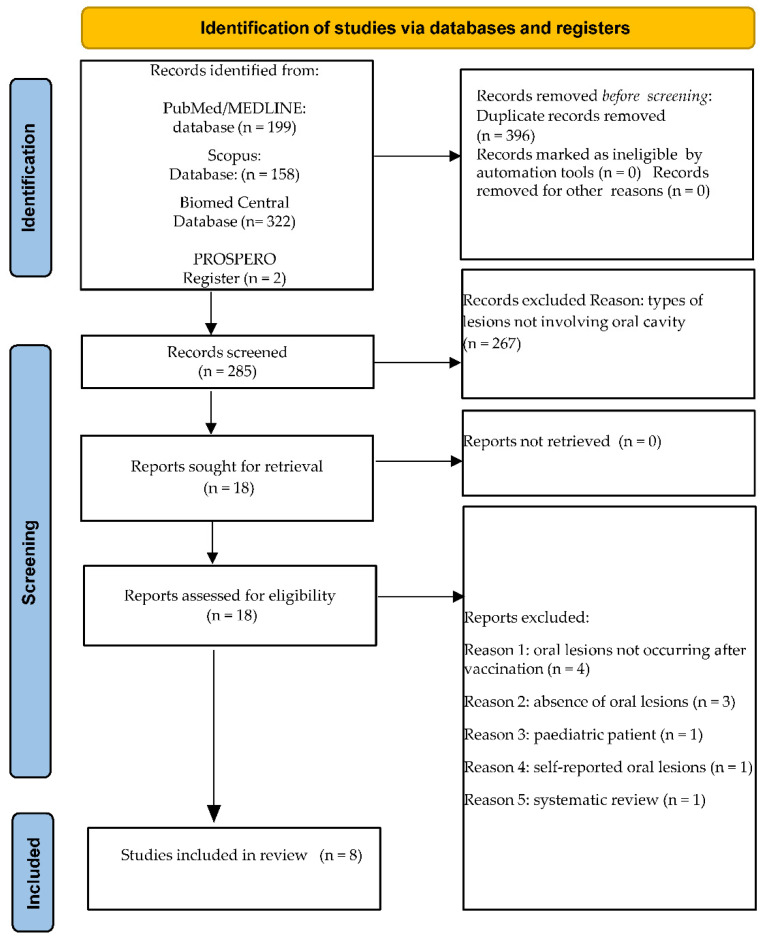
PRISMA 2020 flow diagram for new systematic reviews, which included searches of databases and registers only.

Figure 1 illustrates the study selection flowchart for electronically retrieved records.

Five additional records [18,19,20,21,22], manually retrieved through the reference lists of the eight articles already included in the present systematic review [9,23,24,25,26,27,28,29] and compliant with currently applied eligibility criteria were considered for the current systematic review.

Finally, 13 articles concerning oral lesions diagnosed through objective examination in adult (≥18 years) subjects who received at least one dose of the WHO Emergency Use Listing approved [10] and EMA authorized [11] anti-SARS-CoV-2 vaccines were included in the present systematic review.

All full-texts were freely available, so it was not necessary to provide any contact with the Authors.

### 3.2. Study Characteristics and Qualitative Synthesis

Of the 13 included studies, 11 were case reports and 2 were case series describing 16 cases diagnosed with post-vaccine SARS-CoV-2 oral lesions.

Table 1 shows the characteristics of the included studies related to source and study methods, along with the qualitative synthesis of the oral outcomes investigated, classifying primary oral lesions [16] and describing reported diagnosis, therapy, and progression of the oral lesions reported. Only data compliant with inclusion/exclusion criteria were extracted and synthesized in Table 1; therefore, those concerning subjects <18 years old were not detailed.

The study population comprised 11 females and 5 males between 20 and 60 years old, with a mean age of 46.63. Comorbidities and related treatments, reported in 5 cases, were: Leiden factor V mutation in a 31 year old female taking oral contraceptives [18]; diabetes mellitus and hypertension, treated with amlodipine, teneligliptin, and metformin in a 60-year-old male [20]; benign pemphigoid of mucous membranes [29] and celiac disease [29] in a 55- and a 20-year-old female, respectively; and a 58-year-old female, under sertraline, lorazepam, atorvastatin, metamizole, and penicillin [9]. Eight participants had a negative history of COVID-19 [9,18,21,25,29], while in one case, the subject became positive for SARS-CoV-2 in the time between the first and second vaccine dose [23]; in the remaining 7 cases, the previous history of COVID-19 was not specified [19,20,21,22,24,26,27,28].

Out of the 16 cases diagnosed with oral lesions following anti-SARS-CoV-2 vaccination, 9 cases were diagnosed with oral lesions following the administration of the Pfizer-BioNTech vaccine [19,24,25,26,28,29], 2 cases after vaccination with the Moderna vaccine [9,27], and 1 case was described after AstraZeneca [18], Serum Institute of India [20], Sinopharm Beijing [23], and Johnson & Johnson [22] vaccines, respectively. Oral lesions occurred consequent to the single dose of the Johnson & Johnson vaccine in one case [22], after the administration of the first dose in a total of 8 cases (5 after the Pfizer-BioNTech [19,26,28,29], and 1 after the AstraZeneca [18], Serum Institute of India [20], and Sinopharm Beijing [23] vaccines, respectively) and in 7 cases after the administration of thesecond dose (4 after the Pfizer-BioNTech, 2 after Moderna, and 1 after Sinopharm Beijing vaccines, respectively) [9,24,25,26,27,29]. In one case [21], data concerning vaccine type and dose were missing. No relation between the vaccine type nor dose administered and oral lesion occurrence was detected. Time to oral lesions onset ranged between 1 and 30 days, with a mean onset timing of 9.41 days.

**Figure 2:**
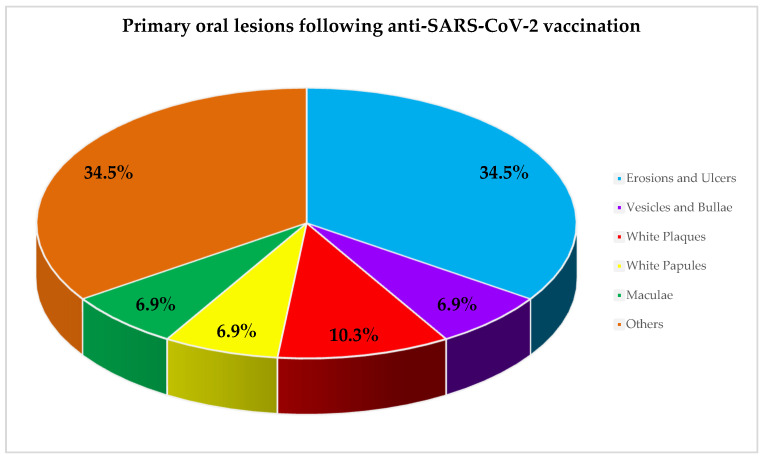
Frequency of reported oral lesions following anti-SARS-CoV-2 vaccination.

Primary oral lesions [16] were (Figure 2): erosions and ulcers in 34.5% (*n* = 10) of the cases, with an erythema multiforme-like pattern (13.8%, *n* = 4) and unspecified (20.7%, 6) patterns, white plaques in 10.3% (*n* = 3) of the cases, and vesicles and bullae observed, similar to erythematous maculae, in 6.9% (*n* = 2) of the cases. Other lesions were described in 34.5% (*n* = 10) of the remaining cases, with no specific reported features (10.35%), appearing as hemorrhagic crusts (10.35%), white papules, swelling, and epithelial desquamation (6.9%).

**Figure 3:**
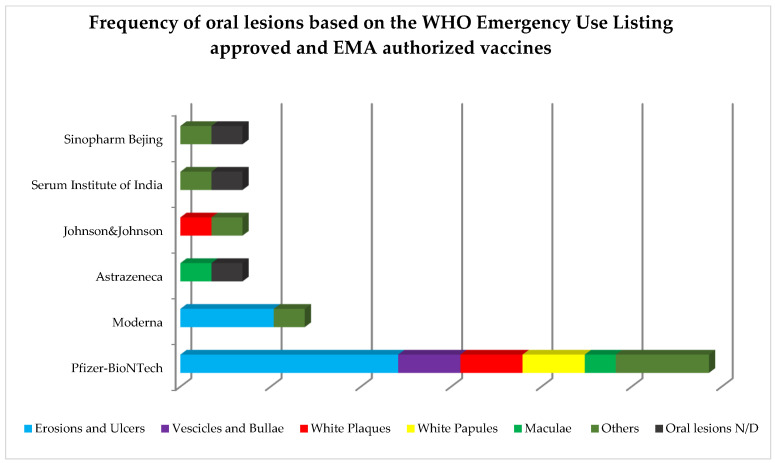
Frequency of oral lesions based on WHO Emergency Use Listing approved and EMA authorized vaccines.

Frequency of oral lesions based on the WHO Emergency Use Listing approved and EMA authorized vaccines is shown in Figure 3.

Diagnostic procedures performed were reported in 12 studies [9,18,19,20,21,22,23,24,25,26,27,28] and comprised: (*n* = 8) serological tests, altogether for herpes simplex type 1 and 2, Epstein Barr, human immunodeficiency, hepatitis B and C viruses, cytomegalovirus, mycoplasma pneumoniae, chlamydia pneumoniae, and treponema pallidum; (*n* = 1) Nucleic Acid Amplification Test (NAAT); (*n* = 2) real-time reverse transcription-polymerase chain reaction (RT-PCR); (*n* = 1) nasopharyngeal swab; 8 biopsies; 1 case of removal of amalgam restorations; (*n* = 1) direct immunofluorescence test; (*n* = 1) skin allergy test; and (*n* = 2) maneuvers for evaluation of Nikolsky’s sign. Histological examination was performed in only fourstudies [21,22,25,27].

**Figure 4:**
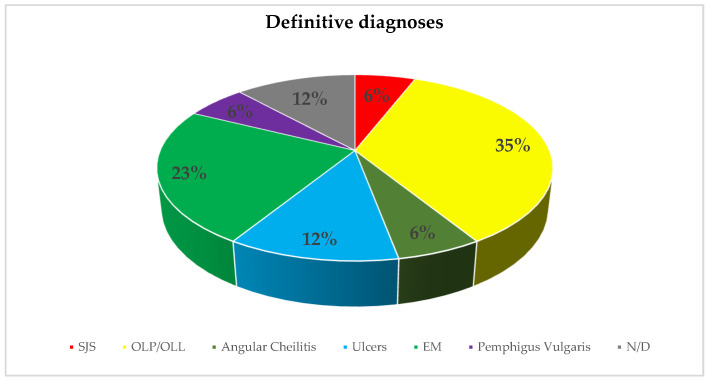
Definitive diagnoses.

Definitive diagnoses reported in the studies currently considered are depicted in Figure 4.

### 3.3. Quality Assessment

Most of the studies were judged at low [19] or moderate [18,23,24,25,26] risk of bias, and 1 study was characterized by a severe [20] risk of bias (Table S1 is available as Supplementary Data), mainly due to missing data, measurement of outcomes, and selection of the reported results.

## 4. Discussion

The present systematic review primarily aimed to assess current data on cases diagnosed with oral lesions among adult subjects (≥18 years old) who had received at least one dose of the World Health Organization (WHO) Emergency Use Listing approved [10] and European Medicines Agency (EMA) authorized [11] anti-SARS-CoV-2 vaccines, classifying oral mucosal manifestations based on primary oral lesions and describing their macroscopic and microscopic features. Secondarily, the possible relationships between such oral lesions and cases’ age, gender, comorbidities, and history of COVID-19, as well as vaccine type and doses administered, were evaluated.

The number of reported cases, 16 in total, diagnosed with oral lesions following anti-SARS-CoV-2 vaccines may seem small, considering that approximately 5.28 billion people, equal to about 68.8% of the world population, received at least one dose of an anti-SARS-CoV-2 vaccine [30,31]. This finding may be due to the paucity of records on oral lesions following anti-SARS-CoV-2 vaccines. In addition, healthcare workers, often committed to severe systemic reactions, which require non-deferrable interventional procedures, such as Stevens–Johnson syndrome, may have paid less attention to the oral cavity.

Moreover, the present systematic review specifically focused on oral lesions, thus excluding other orofacial ADRs, described following the mRNA BNT162b2 Comirnaty (Pfizer-BioNTech) and mRNA-1273 Spikevax (Moderna) vaccines [3]. These ADRs comprise severe, albeit rare (more than 1 in 1000 subjects), allergic reactions, causing tongue, lip, and/or face swelling and Bell’s palsy; facial swelling has also been observed after the mRNA-1273 Spikevax (Moderna) vaccine in subjects undergoing cosmetic labial and facial fillers. Considering that relevant data are currently available mainly from European countries, it is conceivable that both oral lesions and overall orofacial manifestations resulting from anti-SARS-CoV-2 vaccination may be even more prevalent.

Furthermore, in ADR cases with multisystemic involvement, oral lesions were frequently described by healthcare workers not specialized in oral medicine, employing very heterogeneous denominations to describe the alterations observed on the oral mucosa [16,32]; this complicated the identification and classification [16] of primary oral lesions. However, oral ADRs following vaccine administration are considered infrequent findings [33,34], and most of the oral lesions detected are frequently coupled with dermatological manifestations, thus attributable to mucocutaneous ADRs [35].

Accordingly, extracted data show a higher incidence of oral lesions following anti-SARS-CoV-2 vaccines in females (68.8%, 11 cases) vs. males (31.2%, 5 cases). Similarly, McMahon et al. [36] described that women had 90% of skin reactions following the Moderna and Pfizer-BioNTech vaccines. In addition, ADRs were found to be more frequent in females than in males, even following other antiviral vaccines (anti-influenza vaccines with inactivated virus; anti-Yellow Fever with a live attenuated virus; anti-morbillus–maricella–rubella; anti-Japanese encephalitis virus) [37]. Thus, females may be considered at a higher risk of experiencing adverse drug reactions [38], also following vaccine administration, secondary to a greater antibody response [39]. Gender differences in body mass index, adipose tissue distribution, and pharmacodynamics may be implicated in such findings [38].

Oral lesions occurred in a cohort of subjects between 20 and 60 years old, with a relatively low mean age of 46.63 years, especially considering that only data from subjects ≥18 years old were included in the analysis. The lack of oral ADRs reported in subjects aged >60 years is in line with the lower incidence of ADRs in subjects aged >65 years, also observed by Chapin-Bardales et al. [40,41]. Correspondingly, more frequent and severe ADRs have been registered among younger subjects, secondary to the greater efficacy of the immune response [40,41]. In particular, after the anti-SARS-CoV-2 vaccination, a higher increase in interferon-γ levels has been identified in younger compared to older subjects, whereas no differences were found following SARS-CoV-2 infection [40,41].

Data concerning comorbidities and related treatments, as well as the history of COVID-19, were severely lacking, thus not allowing a better comprehension of putative relations between cases’ health general status, vaccine administration, and oral lesion occurrence.

The mechanism of action of anti-SARS-CoV-2 vaccines varies according to the WHO Emergency Use Listing approved and EMA authorized vaccine design [42]. Specifically, mRNA BNT162b2 Comirnaty (Pfizer-BioNTech) and mRNA-1273 Spikevax (Moderna) are mRNA-based and delivered through lipid nanoparticles, while Vaxzevria ChAdOx1-S (Astrazeneca), Ad26.COV2.S (Johnson&Johnson), and ChAdOx1 nCoV-19 Covishield (Serum Institute of India) are vaccines with a non-replicating viral vector; Covilo/BBIBP-Corv (Sinopharm Beijing) is based on inactivated virus [42]. Despite acting through different mechanisms, all of the above vaccines share several adverse reactions, potentially developing after the first, the second, or, hypothetically, the booster dose, comprising: pain at the injection site, pyrexia, headache, vomiting, nausea [42] and, less frequently, dermatological manifestations, such as maculopapular or morbilliform cutaneous eruptions, urticaria, varicella-like lesions, and varicella-zoster virus reactivation [43]. In relation to vaccine type, a higher prevalence of cases (9 out of 16 cases) was diagnosed following mRNA BNT162b2 Comirnaty (Pfizer-BioNTech) vaccine [19,24,25,26,28,29], probably as a consequence of the higher number of doses administered in Europe compared to other vaccines [31]. In more detail, in eight cases [18,19,20,22,26,28,29], oral lesions occurred following the first vaccine dose, while in seven cases [9,24,25,26,27,29], they followed the second vaccine dose; in one case, data were missing. However, no relation between the occurrence of oral ADRs and the number of administered doses, in agreement with Mazur et al. [35], reporting that 3.1% of oral ADRs followed the first and 4% the second dose of vaccine administration.

Oral lesions were detected within 1 to 30 days after vaccination, with a mean onset time of 9.41 days. Therefore, applying the classification proposed for mucocutaneous manifestations following anti-SARS-CoV-2 vaccination [38], which identify acute (<24 h) and delayed (>24 h) manifestations, all lesions should be considered as delayed ADRs. Similarly, Hatami et al. [38] concluded that most frequently reported mucocutaneous adverse effects were delayed-type hypersensitivity reactions, and occurred following the mRNA-1273 Spikevax (Moderna)vaccine [38], thus suggesting its higher immunogenicity when compared to the mRNA BNT162b2 Comirnaty (Pfizer-BioNTech) vaccine. However, our results are in contrast with such a hypothesis, since most of the cases currently analyzed were diagnosed following the mRNA BNT162b2 Comirnaty (Pfizer-BioNTech) vaccine. The lack of acute [38] oral lesions (<24 h) may be partially explained by the current paucity of cases reported in the literature and is likely attributable to the short (24 h) post-vaccination time interval in which the patient may become aware of the appearance of a lesion in the oral cavity, especially if not erosive/ulcerative patterned, and thus, asymptomatic. Conversely, cutaneous ADRs may be detected much more effortlessly, albeit still asymptomatic, even through self-assessment by visual inspection.

The most prevalent primary oral lesions found in adult patients (≥18 years) receiving at least one dose of the anti-SARS-CoV-2 vaccine were erosions and ulcers (34.5%), similar to the primary oral lesions most frequently encountered (48.96%) in SARS-CoV-2-positive adult (≥18 years) subjects [16,44]. Specifically, a higher prevalence was found in erythema-multiforme-like erosive-ulcerative lesions in the vaccinated (16.76%) when compared to ill (1.07%) subjects, while aphthous-like and herpetiform patterns, described in COVID-19 [16], were not observed following vaccine administrations. It may be speculated that the higher prevalence of erosions and ulcers found both in COVID-19 and following anti-SARS-CoV-2 vaccination could be related to vasculitic phenomena and (micro)thrombotic events, secondary to the activation of the immune-inflammatory response, determining, in turn, partial or total occlusion of the small and medium caliber vessels of the chorion of the oral mucosa [45], and the related erosive/ulcerative phenotype. It is worth noting that the presented results clearly highlight that all erosions and ulcers occurred following the mRNA BNT162b2 Comirnaty (Pfizer-BioNTech) (*n* = 7) and the mRNA-1273 Spikevax (Moderna) (*n* = 3) vaccines. This finding, if validated, would suggest that the erosive-ulcerative phenotype may be the most frequently associated with the mRNA-based vaccine design.

Other oral lesions recorded in descending order of frequency were those lacking a suitable definition (10.35%), hemorrhagic crusts (10.35%), white plaques (10.3%), swelling (6.9%), white papules (6.9%), maculae (6.9%), scaling (6.9%), and vesicles and bullae (6.9%). Noteworthy, no cases were diagnosed with necrotizing periodontal disease, which was reported in 0.63%-1.27% of SARS-CoV-2 positive cases, thus reinforcing the hypothesis that such manifestations may be the expression of patients’ reduced immune capacity and concurrent oral dysbiotic phenomena occurring in COVID-19 [16], rather than implicated in oral ADRs.

Similar to other vaccines, such as those against Clostridium tetani, Corynebacterium diphtheria, Bordetella pertussis, measles, mumps, rubella, influenza, and, especially, hepatitisB [23,29], the most definitive diagnoses recorded were oral lichen planus or lichenoid lesions and erythema multiforme. However, the histopathological analysis was severely underreported, therefore it may be assumed that diagnosis mainly relied on clinical features.

Despite applying very inclusive eligibility criteria, very few studies were retrieved from the literature, specifically case reports and series, which were inherently characterized by low scientific evidence. Moreover, collected data were very heterogeneous and incomplete, precluding meta-analysis and conclusive results, which may constitute the main limitations of this study. However, the present study may be considered the first to analyze and to synthesize available data on oral lesions, potentially occurring in adult (≥18 years old) subjects that received at least one dose of the anti-SARS-CoV-2 vaccine. In addition, the present study could be considered the first to classify oral lesions following anti-SARS-CoV-2 vaccinations based on primary oral lesions, to describe their macroscopic and microscopic features, and to evaluate their possible relationship with cases’ age, gender, comorbidities, and the history of COVID-19, as well as with vaccines’ type andadministered doses.

In addition, although some of the outcomes reported may be considered immune-related activations of a pre-existing autoimmune disease rather than true ADRs, such as those oral lesions described by Petruzzi et al. 2022 [29] in subjects with mucous membrane pemphigoid and celiac disease, the findings currently described should nevertheless be considered as preliminary descriptive data. As a matter of fact, presented data may concomitantly suggest that subjects suffering from autoimmune disorders, as well as those with latent herpes simplex type 1 and varicella zoster virus infections [41] may be at risk of disease exacerbation and infection reactivation following anti-SARS-CoV-2 vaccination. Moreover, even if not focused on the genesis of oral lesions, presented results may indirectly disclose putative pathogenic mechanisms potentially underlying ADRs showing at least a temporal relation with the anti-SARS-CoV-2 vaccine administration [46] and involving the oral mucosa.

A better understanding of cases’ and vaccines’ characteristics affecting the occurrence of oral lesions following anti-SARS-CoV-2 vaccine administration may enhance oral healthcare workers’ awareness and preparedness to comprehensively provide oral and dental care [47,48,49,50,51,52], as well as in interdisciplinary settings [53,54,55,56,57,58,59].

## 5. Conclusions

Data extracted and analyzed in the present systematic review show an overall low prevalence of oral lesions following anti-SARS-CoV-2 vaccination, appearing to be higher in females (68.8%) compared to males (31.2%) and showing a slight predilection for subjects <60 years old. Erosions and ulcers (34.5%), undefined oral lesions (10.35%), hemorrhagic crusts (10.35%), white plaques (10.3%), swelling (6.9%), white papules (6.9%), maculae (6.9%), epithelial desquamation (6.9%), and vesicles and bullae (6.9%) were found following the Pfizer-BioNTech vaccine in 9 cases, the Moderna vaccine in 2 cases, and the AstraZeneca, Serum Institute of India, Sinopharm Beijing, and Johnson&Johnson in 1 case, respectively; in the remaining case, vaccine type was not specified. In 8 cases, the oral lesions occurred following the administration of the first dose and in 7 after the second dose; in 1 case, data were missing.

Given the newly emerging viral variants and the constant evolution of commercially developed vaccines, the presented findings may be rapidly updated. Moreover, further studies should be conducted to estimate the overall prevalence of cases diagnosed with oral lesions among adult subjects undergoing anti-SARS-CoV-2 vaccination and to highlight putative relevant factors affecting ADRs occurrence. Furthermore, future investigations may assess specific clinical phenotypes and histopathological patterns of oral lesions following anti-SARS-CoV-2 vaccination, in relation to cases’ comorbidities and ongoing treatments, as well as to vaccine type and doses administered, thus improving oral and general healthcare.

## Figures and Tables

**Table 1:** Data extracted and collected from the studies included in the present systematic review. Studies: first Author, year, and journal of publication; study design, reference number, and funding. Methods: participants’ sample size (n.), mean age (y.o.), gender ratio (M/F), comorbidities, ongoing treatments, and history of COVID-19; intervention. Intervention: vaccine type, dose (1st, 2nd, booster), and time to oral lesions onset. Macroscopic and microscopic features of reported primary oral lesions: Erosions and Ulcers (Aphthous-like “Apht.”, Erythema Multiforme-like “EM”, Herpetiform “Herp.”, Plaques (White, Red), Vesicles and Bullae, Maculae and Petechiae, Others; Number (Single/Multiple); Distribution (Unilateral/bilateral asymmetrical or symmetrical); Location; Cyto/histopathology. Macroscopic and microscopic features of reported other oral lesions. Diagnosis, therapy, and progression of oral lesions: reported diagnosis (OLP, Pemphigus, EM, SJS, Ulcers, Others); diagnostic procedure(s) performed (any); therapy (any), progression (any).

Studies	Population	Anti-SARS-CoV-2 Vaccine	Primary Oral Lesions	Other Oral Lesions	Diagnosis, Therapy and Progression
**Azzi,** 2021 Oral Dis [18] **Case report ** No funding	*n* = 1 31 y.o. 1F Comorbidities: Heterozygous Factor V Leiden Mutation Ongoing treatments: oral contraceptives History of COVID-19: no	AstraZeneca Vaccine dose: 1st Time to onset: 3 days	**Macula*n* = 1** Number: N.A. Distribution: N.A. Location: buccal mucosa, tongue, gums, palate Cyto/histopathology: N.A.	Swollen red lesions *n* = 1 Number: multiple Distribution: N.A. Location: buccal mucosa, tongue, gums, palate Cyto/histopathology: N.A.	**Diagnosis:N.A.** Diagnostic procedure(s): NAAT (-) Serological texts (N.A.) Therapy: Topical Betamethasone (effervescent tablets, 1 mg 3/day) Topical miconazole (oral gel, 2%) Progression: healed after 3d
**Babazadeh,** 2022 Clin Case Rep [23] **Case report** No funding	*n* = 1 52 y.o. 1F Comorbidities: none Ongoing treatments: N.A. History of COVID-19: after first vaccine dose	Sinopharm Bejing Vaccine dose: 1st, 2nd Time to onset: 7–14 days, N.A.		N.A. lesions *n* = 1 Number: multiple Distribution: N.A. Location: N.A. Cyto/histopathology: N.A. Desquamation *n* = 1 Number: multiple Distribution: N.A. Location: lips Cyto/histopathology: N.A.	**Diagnosis:OLP***n* = 1 Diagnostic procedure(s): Serological texts (-HBV, HCV, HIV; +RT-PCR) Therapy: Prednisone (N.A.) Progression: healed after a few days from the 1st dose; more acute reappearance after the 2nd dose
**Borg,** 2022 JEADV [19] **Case report** No funding	*n* = 1 38 y.o. 1M Comorbidities: none Ongoing treatments: none History of COVID-19: N.A.	Pfizer-BioNTech Vaccine dose: 1st Time to onset: 2 days	**Erosions and Ulcers*n* = 1** EM (*n* = 1) Number: single Distribution: unilateral Location: hard palate Cyto/histopathology: N.A.		**Diagnosis:EM***n* = 1 Diagnostic procedure(s): Nikolsky sign (-) Biopsy (bulla on the left forearm) Therapy: Prednisone (40 mg/die for 5d) Progression: healed after 7d
**Caggiano,** 2022 Oral Dis [24] **Case report** No funding	*n* = 1 40 y.o. 1M Comorbidities: N.A. Ongoing treatments: N.A. History of COVID-19: N.A.	Pfizer-BioNTech Vaccine dose: 2nd Time to onset: 30 days	**Plaques*n* = 1** White (*n* = 1) Number: multiple Distribution: bilateral symmetrical Location: cheeks Cyto/histopathology: N.A.		**Diagnosis:OLP***n* = 1 Diagnostic procedure(s): Serological texts (↓MCHC = 31,6 g/dL; ↑ PCR = 0,57 mg/dL) Amalgam fillings removal Incisional biopsy Therapy: N.A. Progression: N.A.
**Calabria,** 2022 Path Res Pract [25] **Case report** No funding	*n* = 1 60 y.o. 1F Comorbidities: N.A. Ongoing treatments: N.A. History of COVID-19: no	Pfizer-BioNTech Vaccine dose: 2nd Time to onset: 7d	**Erosions*n* = 1 and Ulcers *n* = 1** Number: multiple Distribution: unilateral Location: lower lip, upper vermillion; fornix; marginal gingiva Cyto/histopathology: N.A. **Vesicles and Bullae*n* = 1** Number: multiple Distribution: bilateral Location: lower lip; upper vermillion; oral floor; tongue; upper fornix; alveolar mucosa; marginal gingiva Cyto/histopathology: “partially ulcerated mucosa covered with only one or more layers of keratinocytes aligned along the basement membrane; at one edge of the biopsy, the non-keratinizing squamous cell epithelium showed severe acantholysis, forming a suprabasal blister with a row of “gravestone” looking basal cells attached to the connective tissue; there was a moderate band-like lymphocytic infiltrate in the subepithelial chorion, with some eosinophils and several small vessels”		**Diagnosis:Pemphigus Vulgaris***n* = 1 Diagnostic procedure(s): Biopsy (peri-lesional on the mandibular gingiva) DIF Serological texts (↑ anti-Dsg-3 antibodies = 80 U/mL; ↑ anti-Dsg-1 antibodies = 4.4 U/mL) Therapy: Prednisone (1 mg/kg for 6 weeks) Rituximab (1000 mg twice at 2wks intervals) Progression: improved within 3wks
**Dash,** 2021 Clin Exp Dermatol [20] **Case report** No funding	*n* = 1 60 y.o. 1M Comorbidities: diabetes, hypertension Ongoing treatments: teneligliptin, metformin, amlodipine History of COVID-19: N.A.	Serum Institute of India Vaccine dose: 1st Time to onset: 3 days		N.A. oral lesions Number: multiple Distribution: N.A. Location: N.A. Cyto/histopathology: N.A. Hemorrhagic crusts *n* = 1 Number: multiple Distribution: N.A. Location: lip Cyto/histopathology: N.A.	**Diagnosis**:**SJS***n* = 1 Diagnostic procedure(s): Biopsy (skin lesions) Therapy: Paracetamol Levocetrizine (N.A.) Ciclosporin (300 mg) Progression: healed after 7d from the start of treatment with Ciclosporin
**Hertel,** 2022 Vaccines [26] **Case series** This work was supported by TRR295, KFO339 (RP)	*n* = 2 53.5 y.o. 1 M 50 y.o.; 1 F 57 y.o. Comorbidities: N.A. Ongoing treatments: N.A. History of COVID-19: N.A.	Pfizer-BioNTech Vaccine dose: 1st, 2nd Time to onset: 9 days, 14 days	**Plaques*n* = 2** Number: multiple Distribution: bilateral symmetrical Location: cheeks; vestibule Cyto/histopathology: N.A.	White papules Number: multiple Distribution: bilateral symmetrical Location: cheeks; vestibule Cyto/histopathology: N.A.	**Diagnosis:OLP***n* = 2, **OLL** *n* = 2 Diagnostic procedure(s): Biopsy (N.A.) Therapy: N.A. Progression: N.A.
**Maeda,** 2022 J Stomatol Oral Maxillofac Surg [27] **Case report** No funding	*n* = 1 58 y.o. 1F Comorbidities: none Ongoing treatments: none History of COVID-19: N.A.	Moderna Vaccine dose: 2nd Time to onset: 20 days	**Erosions and Ulcers*n* = 2** Number: multiple Distribution: bilateral symmetrical Location: hard palate Cyto/histopathology: “nonspecific ulcer without caseous necrosis; there were no signs of a tumor; increased levels of local T helper type 1 cytokine (e.g., interferon-γ) production”		**Diagnosis:Ulcers***n* = 2 Diagnostic procedure(s): Biopsy (left ulcer) PAS-reaction (- IHC (-HIV-1, CMV, EBV) Serological texts (↓ white blood cells, C-reactive protein; -desmoglein-1 and -3 antigens, bullous pemphigoid-180 antigen, HIV antigen, rapid plasma reagin, tuberculosis; ↑ Th1lymphocytes cytokines) Nikolsky sign (-) Therapy: Acetaminophen (600 mg/die for 7 d) Topical lidocaine (4%) Topical unspecified steroid (ointment) Sodium azulene sulfonate (mouthwash) Progression: healed after 7 d
**Manfredi,** 2021 Oral Dis [28] **Case report** No funding	*n* = 1 34 y.o. 1F Comorbidities: none Ongoing treatments: none History of COVID-19: no	Pfizer-BioNTech Vaccine dose: 1st Time to onset: 2 days	**Erosions and Ulcers*n* = 1** Number: multiple Distribution: N.A. Location: oral floor, lips, gingiva Cyto/histopathology: N.A. **Maculae and Petechiae*n* = 1** Erythema *n* = 1 Number: multiple Distribution: N.A. Location: tongue Cyto/histopathology: N.A.	Swelling *n* = 1 Location: lips, gingiva Cyto/histopathology: N.A.	**Diagnosis:Ulcers***n* = 1, **Angular cheilitis** *n* = 1 Diagnostic procedure(s): Allergological cutaneous tests (+Polysorbato 80 andglicopolyethilene) Therapy: Topical antibacterial agents (N.A.) Moisturizing lip balm Progression: healed after 10–15d
**Petruzzi,** 2022 BMC Oral Health [29] **Case series** No funding	*n* = 3 41,3 y.o. 3F 55, 49, 20 y.o. Comorbidities: Mucous membrane pemphigoid (MMP)/None/celiac disease Ongoing treatments: N.A. History of COVID-19: no	Pfizer-BioNTech Vaccine dose: 1st, 2nd, 1st Time to onset: 10 days, 1 days, 18 days	**Erosions (EM)*n* = 3** Number: multiple Distribution: N.A. Location: N.A.; oral floor, tongue, gingiva, soft palate; lips, gingiva Cyto/histopathology: N.A. **Vesicles and Bullae*n* = 1** Number: multiple Distribution: N.A. Location: oral floor, tongue Cyto/histopathology: N.A.	Squamous crusted lesions *n* = 2 Number: multiple Distribution: M/D Location: lips; vermillion Cyto/histopathology: N.A.	**Diagnosis:EM***n* = 3 Diagnostic procedure(s): N.A. Therapy: Prednisone (25 mg for 10 days, in 2 Pt.) Oral prednisone (25 mg for 3 weeks, in 1 Pt.) Topical clobetasol propionate (gel 0.05%, in all Pt.) Progression: N.A.
**Saibene,** 2021 Clin Case Rep [9] **Case report** No funding	*n* = 1 58 y.o. 1F Comorbidities: N.A. Ongoing treatments: sertraline, lorazepam, atorvastatin, metamizole, penicillin History of COVID-19: no	Moderna Vaccine dose: 2nd Time to onset: 1 days	**Erosions *n* = 1** Number: multiple Distribution: N.A. Location: N.A. Cyto/histopathology: N.A.	Swelling Location: oral floor Cyto/histopathology: N.A.	**Diagnosis:N.A.** Diagnostic procedure(s): Serological texts (-chlamydia, pneumoniae, mycoplasma pneumoniae, T. pallidum, HHV-1 and HHV-2, HCV, HBV; +HBsAb = 438 UI/L, HHV IgG = 22.1 titration index; ↑ PCR = 23.9 mg/L) Nasopharyngeal swab (-) Therapy: Methyl-prednisolone (1 mg/kg for 5 d) Morphine (for 48 h) Fluid supplementation Progression: N.A.
**Sharda,** 2022 JEADV [21] **Case report ** No funding	*n* = 1 35 y.o. 1F Comorbidities: none Ongoing treatments: N.A. History of COVID-19: no	N.A. Vaccine dose: N.A. Time to onset: 14 days		“Erythematous base with white reticular streaks over them, some had erosions” Number: multiple Distribution: bilateral symmetrical Location: cheeks, gums Cyto/histopathology: “Moderately dense superficial perivascular lichenoid infiltrate of lymphocytes and plasma cells with irregular acanthosis and vacuolation of the basal layer. The dermo-epidermal junction is focally infiltrated by lymphocytes and shows scattered necrotic keratinocytes”	**Diagnosis:OLP***n* = 1 Diagnostic procedure(s): Biopsy RT-PCR (-) Serological texts (-HBV, HCV, HIV) Therapy: N.A. Progression: N.A.
**Troeltzsch,** 2021 Oral Dis [22] **Case report** No funding	*n* = 1 49 y.o. 1M Comorbidities: N.A. Ongoing treatment: N.A. History of COVID-19: N.A.	Johnson & Johnson Vaccine dose: N.A. Time to onset: 6 days	**Plaques(white) *n* = 1** Number: multiple Distribution: N.A. Location: cheeks, tongue Cyto/histopathology: “Linear accumulation of lymphocytes along the basal epidermal membrane with intraepidermal lymphocytic infiltrates and single necrotic keratinocytes”	Desquamations Number: multiple Distribution: N.A. Location: N.A. Cyto/histopathology: N.A.	**Diagnosis:OLP** n.1 Diagnostic procedure(s): Biopsy (N.A.) Therapy: Topical clobetasol (oral irrigation 0.5 mg/mL for 4 weeks) Progression: N.A.

Abbreviations: number, “n.”, years old, “y.o.”; day(s), “d”; week(s), “wk(s)”; Vaxzevria ChAdOx1-S (AstraZeneca), “AstraZeneca”; Covilo/BBIBP-Corv (Sinopharm Beijing), “Sinopharm Beijing”; mRNA BNT162b2Comirnaty (Pfizer-BioNTech), “Pfizer-BioNTech”;ChAdOx1 nCoV-19 Covishield (Serum Institute of India), “Serum Institute of India”; mRNA-1273 Spikevax (Moderna), “Moderna”; Aphthous-like, “Apht.”; Erythema Multiforme-like, “EM”; Herpetiform, “Herp.”; Oral Lichen Planus, “OLP”; Oral Lichenoid Lesions, “OLL”; Stevens-Johnson Syndrome, “SJS”; increased value, “↑”; decreased value, “↓”; positive for, “+”; negative for, “-“; Nucleid Acid Amplification Tests, “NAATs”; Real-time reverse Transcription–Polymerase Chain Reaction, “RT-PCR”; not available, “N.A.”; Direct immunofluorescence, “DIF”; Immunohistochemistry, “IHC”; patients, “Pt”.

## Data Availability

Data are available on Scopus, MEDLINE/PubMed, and BioMed Central databases, and on the PROSPERO register.

## Supplementary Materials

The following supporting information can be downloaded at: https://www.mdpi.com/article/10.3390/ijerph191610228/s1, Table S1: Risk of bias of the studies included in the systematic review: Y = low risk of bias, PY = moderate risk of bias, PN = serious risk, N = critical risk of bias, NI = no information available.
