# Supplementary material for: Oral Lesions Following Anti-SARS-CoV-2 Vaccination: A Systematic Review
Source: Int J Environ Res Public Health. 2022 Aug 17;19(16):10228. doi: 10.3390/ijerph191610228 (PMC9408767; doi:10.3390/ijerph191610228)
Supplement: Supplementary file 1 [file ijerph-19-10228-s001.zip › ijerph-1862343-supplementary.pdf]

Table S1. Risk of bias of the studies included in the systematic review: Y = low risk of bias, PY = moderate risk of bias, PN = serious risk, N = critical risk of bias, NI = no information available.

Studies	Confounding	Selection of participants	Measurement classification of interventions	Deviations from intended interventions	Missing data	Measurement of outcomes	Selection of the reported results
**Azzi,** 2021 [17]	Y / **PY** / PN / N/ NI	**Y** / PY / PN / N/ NI	**Y** / PY / PN / N/ NI	**Y** / PY / PN / N/ NI	Y / PY / PN / **N**/ NI	Y / PY / PN / **N**/ NI	Y / **PY** / PN / N/ NI
**Babazadeh,** 2022 [22]	Y / **PY** / PN / N/ NI	**Y** / PY / PN / N/ NI	**Y** / PY / PN / N/ NI	**Y** / PY / PN / N/ NI	Y / **PY** / PN / N/ NI	Y / **PY** / PN / N/ NI	Y / **PY** / PN / N/ NI
**Borg,** 2022 [18]	**Y**/ PY / PN / N/ NI	**Y** / PY / PN / N/ NI	**Y** / PY / PN / N/ NI	**Y** / PY / PN / N/ NI	**Y** / PY / PN / N/ NI	**Y** / PY / PN / N/ NI	**Y** / PY / PN / N/ NI
**Caggiano,** 2022 [23]	Y / **PY** /PN / N/ NI	**Y** / PY / PN / N/ NI	**Y** / PY / PN / N/ NI	**Y** / PY / PN / N/ NI	Y / **PY** / PN / N/ NI	Y / **PY** / PN / N/ NI	Y / **PY** / PN / N/ NI
**Calabria,** 2022 [24]	Y / **PY** / PN / N/ NI	**Y** / PY / PN / N/ NI	**Y** / PY / PN / N/ NI	**Y** / PY / PN / N/ NI	Y / **PY** / PN / N/ NI	**Y** / PY / PN / N/ NI	**Y** / PY / PN / N/ NI
**Dash, S.** 2021 [19]	Y / PY / **PN** / N/ NI	**Y** / PY / PN / N/ NI	**Y** / PY / PN / N/ NI	**Y** / PY / PN / N/ NI	Y / **PY** / PN / N/ NI	**Y** / PY / PN / N/ NI	Y / **PY** / PN / N/ NI
**Hertel,** 2022 [25]	Y / **PY** / PN / N/ NI	**Y** / PY / PN / N/ NI	**Y** / PY / PN / N/ NI	**Y** / PY / PN / N/ NI	Y / **PY** / PN / N/ NI	**Y** / PY / PN / N/ NI	Y / **PY** / PN / N/ NI
**Maeda,** 2022 [26]	**Y** / PY / PN / N/ NI	**Y** / PY / PN / N/ NI	**Y** / PY / PN / N/ NI	**Y** / PY / PN / N/ NI	**Y** / PY / PN / N/ NI	**Y** / PY / PN / N/ NI	**Y** / PY / PN / N/ NI
**Manfredi,** 2021 [27]	**Y** / PY / PN / N/ NI	**Y** / PY / PN / N/ NI	**Y** / PY / PN / N/ NI	**Y** / PY / PN / N/ NI	Y / **PY** / PN / N/ NI	Y / **PY** / PN / N/ NI	**Y** / PY / PN / N/ NI
**Petruzzi,** 2022 [28]	Y / PY / **PN** / N/ NI	**Y** / PY / PN / N/ NI	**Y** / PY / PN / N/ NI	**Y** / PY / PN / N/ NI	Y / PY / **PN** / N/ NI	Y / PY / **PN** / N/ NI	Y / PY / **PN** / N/ NI
**Saibene,** 2021 [9]	Y / **PY** / PN / N/ NI	**Y** / PY / PN / N/ NI	**Y** / PY / PN / N/ NI	**Y** / PY / PN / N/ NI	Y / **PY** / PN / N/ NI	Y / **PY** / PN / N/ NI	Y / **PY** / PN / N/ NI
**Sharda,** 2022 [20]	Y / **PY** / PN / N/ NI	**Y** / PY / PN / N/ NI	**Y** / PY / PN / N/ NI	**Y** / PY / PN / N/ NI	**Y** / PY / PN / N/ NI	**Y** / PY / PN / N/ NI	**Y** / PY / PN / N/ NI
**Troeltzsch,** 2021 [21]	Y / **PY** / PN / N/ NI	**Y** / PY / PN / N/ NI	**Y** / PY / PN / N/ NI	**Y** / PY / PN / N/ NI	**Y** / PY / PN / N/ NI	**Y** / PY / PN / N/ NI	**Y** / PY / PN / N/ NI
